# Downregulation of microRNA-23a suppresses prostate cancer metastasis by targeting the PAK6-LIMK1 signaling pathway

**DOI:** 10.18632/oncotarget.2880

**Published:** 2015-01-16

**Authors:** Songwang Cai, Ruihan Chen, Xiaojuan Li, Yi Cai, Zhiqiang Ye, Shigeng Li, Jun Li, Huaiqiu Huang, Shubin Peng, Jun Wang, Yiran Tao, Hongxing Huang, Xinglai Wen, Jianfeng Mo, Zhupeng Deng, Jian Wang, Yangfan Zhang, Xin Gao, Xingqiao Wen

**Affiliations:** ^1^ Department of Cardiothoracic Surgery, The Third Affiliated Hospital, Sun Yat-sen University, Guangzhou, China; ^2^ Department of Emergency, The Third Affiliated Hospital, Sun Yat-sen University, Guangzhou, China; ^3^ Department of Health Care, The Third Affiliated Hospital, Sun Yat-sen University, Guangzhou, China; ^4^ Department of Urology, The Third Affiliated Hospital, Sun Yat-sen University, Guangzhou, China; ^5^ Department of Dermatology, The Third Affiliated Hospital, Sun Yat-sen University, Guangzhou, China; ^6^ Department of Urology, Zhongshan People's Hospital, Zhongshan City, Guangdong, China; ^7^ Department of Urology, Qingyuan People's Hospital, Qingyuan City, Guangdong, China; ^8^ Department of Urology, Taishan People's Hospital, Taishan City, Guangdong, China; ^9^ Department of Urology, The First People's Hospital of Foshan City, Foshan City, Guangdong, China

**Keywords:** microRNA, miR23a, prostate cancer, metastasis, cytoskeleton

## Abstract

Here we found that levels of miR-23a were decreased in prostate cancer cell lines and tumor tissues. These low levels were associated with poor patients' prognosis. MiR-23a inhibited migration and invasion of prostate cancer *in vivo* and in orthotopic prostate cancer mice model. MiR-23a decreased levels of p21-activated kinase 6 (PAK6). Expression of miR-23a inhibited phosphorylation of LIM kinase 1 (LIMK1) and cofilin, in turn suppressing formation of stress fibers and actin filaments, which was required for cell motility and invasion. PAK6 bound to LIMK1 and activated it via phosphorylation at Thr-508. Also, PAK6 and LIMK1 were colocalized in the cytoplasma. Thus, miR-23a regulated cytoskeleton by affecting LIMK1 and cofilin. In summary, we have identified the miR-23a-PAK6-LIMK1 pathway of prostate cancer metastasis. Potential therapeutic approach by targeting miR-23 is suggested.

## INTRODUCTION

Prostate cancer is the most common malignancy in males and the second leading cause of cancer death among men. Although early-stage prostate cancer is able to be managed, the evolution of prostate cancer to a hormone-independent stage is invariably associated with advanced metastatic disease with limited therapeutic options [1].

MicroRNAs (miRNAs) are a class of endogenously expressed, noncoding small RNAs of approximately 22 nucleotides in length. miRNAs regulate gene expression by targeting protein-encoding mRNA [2]. Recently, many reports have documented a functional contribution of specific miRNAs to the regulation of metastasis in various cancer types [3–8].

In prostate cancer cells, alterations in miRNA expression have been observed, miRNAs may act as key regulators of metastasis activities [9, 10]. Several deregulated miRNAs have been shown to be able to regulate the cell migration and invasion of prostate cancer cells [11–15]. The actin cytoskeleton is a dynamic structure, in which actin polymerization and depolymerization rates control cell motility, cell division, and the formation of specialized structures. However, the signaling of how miRNAs regulate cytoskeletal changes in prostate cancer remains not fully understood.

In this study, an expression profiling of human miRNAs in paired prostate cancer and adjacent non-tumor tissues was performed. MiR-23a was identified to be associated with a metastatic and poor prognostic phenotype. Ectopic overexpression of miR-23a in prostate cancer cells resulted in inhibiting invasion and metastasis abilities both *in vitro* and *in vivo*. Additional analyses revealed that miR-23a directly targeted the p21-activated kinase 6 (PAK6) gene to suppress the phosphorylation of LIM kinase 1 (LIMK1), resulting in cytoskeletal reorganization, and ultimately, the inhibition of prostate cancer cell invasion and metastasis.

MiR-23a-PAK6-LIMK1 was showed to be a novel regulatory pathway that contributed to prostate cancer metastasis. Our findings also suggest treatment targeting miR-23 have potential benefit for patients with prostate cancer.

## RESULTS

### MicroRNA expression profiling in prostate cancer

**Figure 1:**
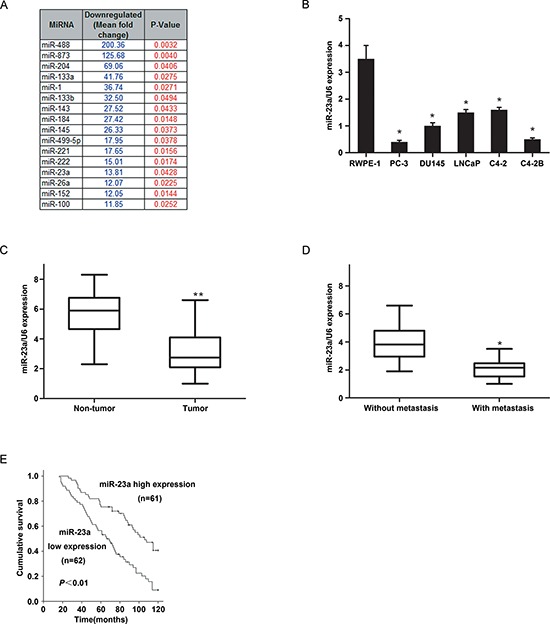
The expression of MiR-23a in prostate cancer cell lines and tissues and its prognostic values in patients **(A)** MicroRNA expression profiling in prostate cancer were examined. 16 mRNAs with at least 10-fold expression down-regulated change were identified as altered markedly in prostate cancer (*p* < 0.05). **(B)** miR-23a expression were examined by real-time PCR in RWPE-1 cells and 5 prostate cancer cell lines (*n* = 3 replicate experiments; *p* < 0.05 compared with control). **(C)** miR-23a expression in 20 paired prostate cancer and adjacent non-tumour tissues. **(D)** miR-23a expression in ten primary and metastatic prostate cancer samples. **(E)** Kaplan-Meier analysis of survival times of patients with prostate cancer as a function of miR-23a levels.

An expression profiling of human miRNAs in three pairs of primary human prostate cancer and matched adjacent non-tumor tissues was performed. In total, 51 miRNAs were identified to be significantly altered in prostate cancer cells (*P* < 0.05, Supplementary Table 1), including 16 ones that were downregulated by at least 10-fold (Figure 1A). Among these 16 miRNAs, 9 have been characterized as tumor suppressors in prostate cancer cells. MiR-23a, which was one of the other 7 miRNAs, is down-regulated significantly. It was reported to promote gliomagenesis [16] and neuroblastoma cell metastasis [17], facilitates mammary and colorectal carcinoma cell invasion and hepatic metastasis [18, 19], suppresses apoptosis and enhances proliferation in hepatocellular carcinoma [20, 21]. Down-regulated miR-23a was consistent with the study analyzing microRNA profiling of prostate cancer [22].

### Decreased miR-23a expression was frequently detected in prostate cancer cells and human prostatic cancer tissues

MiRNA expression levels were examined by real-time PCR in six prostate cell lines and in 20 paired human prostate cancer and matched adjacent non-tumor tissues. The results showed that, all five metastatic prostate cancer cell lines (PC-3, DU145, LNCaP, C2-4 and C4-2B) had lower miR-23a expression than the normal prostate cell line RWPE-1 (Figure 1B). In addition, mean miR-23a expression was significantly lower in the primary prostate cancer samples than that in the matched non-tumor tissues (*P* < 0.01) (Figure 1C and Supplementary Figure 1C). Furthermore, mean miR-23a expression was significantly lower in the ten metastatic prostate cancer samples than that in the primary prostate cancer samples (*P* < 0.01) (Figure 1D).

### Low miR-23a expression was associated with aggressive and poor prognostic prostate cancer phenotype

We further investigated the pathological and prognostic significance of miR-23a levels in patients with prostate cancer. The expression of miR-23a in a cohort of 123 prostate cancer tissues was examined by real-time PCR. The median expression level of all 123 prostate cancer samples was chosen as the cut-off point for separating tumors with low miR-23a expression from those with high expression. Overall, 62/123 prostate cancer samples exhibited low miR-23a expression, whereas 61/123 showed high expression (Table 1). The correlation analysis revealed that low miR-23a expression in prostate cancer was associated with a more aggressive tumor phenotype (*P* < 0.05, Table 1, Figure 1D). The Kaplan-Meier analysis revealed that low miR-23a expression in prostate cancer was associated with decreased survival time (*P* < 0.05, Table 2, Figure 1E). An additional multivariate Cox regression analysis indicated that low miR-23a expression was an independent prognostic factor for poor survival in patients with prostate cancer (*P* = 0.002, Table 2).

**Table 1:** Correlation of miR-23a expression in tissues with clinicopathological variables of patients in 123 cases of prostate cancer

Variables	miR-23a			*p* Value
	All cases (*n* = 123)	Low expression (*n* = 62)	High expression (*n* = 61)	
Age(years)				
≤ 68.5	66	32 (48.5)	34 (51.5)	0.646
> 68.5	57	30 (52.6)	27 (47.4)	
PSA level (μg/L)				
≤ 10	64	23 (35.9)	41 (64.1)	0.001
> 10	59	39 (66.1)	20 (33.9)	
gleason				
≤ 7	93	42 (45.2)	51 (54.8)	0.041
> 7	30	20 (66.7)	10 (33.3)	
Distant metastasis				
M0	111	52 (46.8)	59 (53.2)	0.016
M1	12	10 (83.3)	2 (16.7)	
Pathologic stage				
≤ T2	90	39 (43.3)	51 (56.7)	0.010
> T2	33	23 (69.7)	10 (30.3)	

**Table 2:** Uinvariate and multivariate analysis of factors associated with survival time of patients with prostate cancer

Clinical variable	Case number	HR(95% CI)	*p* Value
**Uinvariate analysis**			
MiR-23a (low vs high)	62/61	0.389 (0.249–0.608)	0.000
Age (> 68.5 vs 68.5)	57/66	0.976 (0.635–1.501)	0.913
PSA level (> 10 vs ≤ 10)	50/64	1.782 (1.159–2.741)	0.009
Gleason (> 7 vs ≤ 7)	32/91	4.941 (3.096–7.885)	0.000
Distant metastasis (M1 vs M0)	12/111	15.724 (7.829–31.580)	0.000
Pathologic stage (> T2 vs T1)	33/90	2.242 (1.428–3.520)	0.000
**multivariate analysis**			
miR-23a (low vs high)	62/61	1.776 (1.116–2.829)	0.015
Pathologic stage (> T2 vs T1)	33/90	1.724 (1.009–2.946)	0.046
Gleason (> 7 vs ≤ 7)	32/91	4.026 (2.415–6.712)	0.000
Distant metastasis (M1 vs M0)	33/90	7.908 (3.529–17.719)	0.000

### Overexpression of miR-23a suppressed invasion and migration of prostate cancer cells *in vitro*

**Figure 2:**
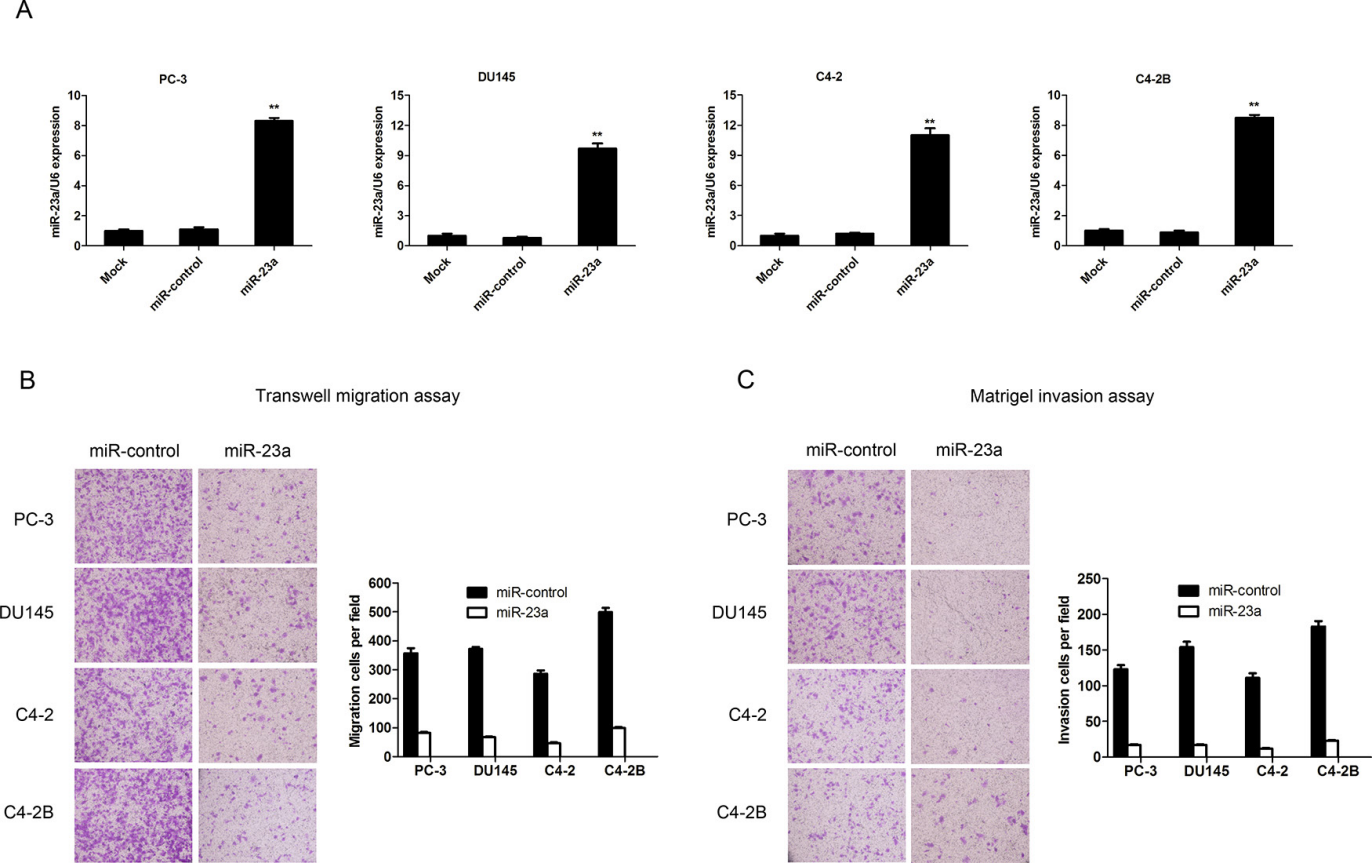
MiR-23a overexpression decreased prostate cancer cell invasion and migration *in vitro* **(A)** Real-time PCR analysis of miR-23a expression in PC-3, DU145, C4-2 and C4-2B cells infected with miR-control-lentivirus or with miR-23a-lentivirus. **(B)** Transwell migration assay with PC-3, DU145, C4-2 and C4-2B cells infected with miR-control-lentivirus or miR-23a-lentivirus. **(C)** Matrigel invasion assay in PC-3, DU145, C4-2 and C4-2B cells infected with miR-control-lentivirus or miR-23a-lentivirus.

To study the potential biological function of miR-23a in prostate cancer cells, we performed transwell migration assays and matrigel invasion assays. MiR-23a expression levels in the PC-3, DU145, C2-4 and C4-2B cells infected with miR-control-lentivirus or with miR-23a-lentivirus were confirmed by real-time PCR (Figure 2A). The overexpression of miR-23a suppressed the migration of the PC-3, DU145, C2-4 and C4-2B cells as evidenced by the transwell migration assays (Figure 2B). The matrigel invasion assays demonstrated that miR-23a overexpression dramatically reduced the invasiveness of the PC-3, DU145, C2-4 and C4-2B cells (Figure 2C).

### MiR-23a inhibited prostate cancer metastasis *in vivo*

**Figure 3:**
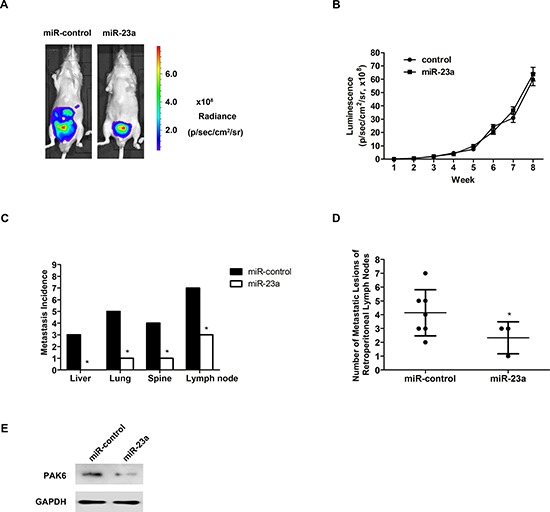
Overexpression of miR-23a suppressed prostate cancer metastasis *in vivo* **(A)** PC-3 cells stably expressing either empty vectors or miR-23a and a luciferase reporter were injected into the prostates of nude mice. Representative bioluminescence images from either control mice (left) or miR-23a-expressing mice (right) were obtained on day 56. **(B)** Quantifications of prostate-associated luciferase activities (*in vivo* bioluminescent imaging) at indicated time points. **(C)** Incidences of metastases in liver, lung, spine and retroperitoneal lymph nodes in the two groups. **(D)** Numbers of metastatic lesions in retroperitoneal lymph nodes in the two groups. **(E)** PAK6 expression in prostate orthotopic tumors were examined by western blotting between two groups.

To assess the effects of miR-23a on tumor metastasis *in vivo*, we generated PC-3 cells that stably expressed luciferase (PC-3-Luc) to enable the live imaging of cancer metastasis using bioluminescent imaging technology. The PC-3-Luc cells engineered to stably express miR-23a or vector controls were then injected into the prostates of immunodeficient mice. Tumor progression was monitored weekly for eight weeks. Representative bioluminescence images of the mice in the control (left) or miR-23a-expression (right) groups on day 56 indicated that miR-23a overexpression decreased the number of metastatic lesions (Figure 3A). The prostate-associated luciferase activity was comparable in the two groups at 8 weeks (Figure 3B), and the prostate orthotopic tumors did not significantly differ between the two groups (*P* > 0.05). An autopsy was performed on the orthotopic model mice to assess the distributions of the metastases. There were fewer metastatic lesions in the miR-23a-expression group than in the control group (Figure 3C, 3D). Furthermore, western blotting analysis demonstrated that PAK6 expression in prostate orthotopic tumors was down-regulated significantly in the miR-23a-expression group compared with the control group (Figure 3E). These results indicated that miR-23a expression in prostate cancer cells significantly suppressed metastasis *in vivo*.

### PAK6 was a direct regulated target of miR-23a

**Figure 4:**
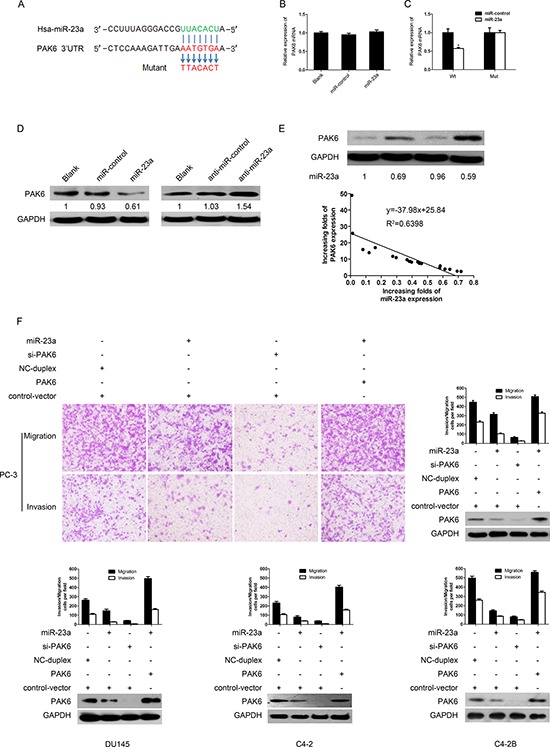
PAK6 is a direct target of miR-23a **(A)** Schematic of predicted miR-23a binding sequence in PAK6 3′-UTR. PAK6 3′-UTR was mutated in complementary site for seed region of miR-23a as indicated. A human PAK6 3′-UTR fragment containing wild-type or mutant miR-23a binding sequence was cloned downstream of luciferase reporter gene. **(B)** Real-time PCR was performed to detect PAK6 expression in PC-3 cells infected with miR-23a-expression vector or with empty vector. Data were normalized to GAPDH mRNA expression. **(C)** Luciferase activity of wild-type (Wt) or mutant (Mut) PAK6 3′UTR reporter gene in PC-3 cells infected with miR-23a-expression vector or empty vector. **(D)** PAK6 immunoblotting in PC-3 cells infected with miR-23a or with control RNA duplex (miR-control) and with anti-miR-23a (miR-23a inhibitor) or with anti-miR-control RNA duplex (negative control). **(E)** miR-23a expression was inversely correlated with PAK6 protein expression in 20 paired prostate cancer and adjacent non-tumor tissues. **(F)** Effects on invasion and migration of PC-3, DU145, C4-2 and C4-2B cells were rescued by overexpressing PAK6, which was consistent with immunoblotting results.

To understand the mechanism by which miR-23a suppressed the migration and invasion of prostate cancer cells, we used target prediction programs (PicTar, TargetScan and miRanda) to predict the targets of miR-23a. PAK6 was identified as a potential target of miR-23a. The 3′-UTR of PAK6 mRNA contained a complementary sequence for the seed region of miR-23a (Figure 4A). MiR-23a overexpression did not elicit the degradation of PAK6 mRNA (Figure 4B).

To determine whether PAK6 was a direct target of miR-23a, a human PAK6 3′-UTR fragment containing a wild-type or a mutant miR-23a binding sequence was cloned downstream of the firefly luciferase reporter gene (Figure 4C). MiR-23a overexpression reduced the activity of a luciferase reporter gene fused to the wild-type PAK6 3′-UTR (43% reduction, *P* < 0.05). Conversely, when we performed luciferase assays using a plasmid harboring a mutant version of the PAK6 mRNA 3′-UTR (the miR-23a binding sites were inactivated by site-directed mutagenesis), the luciferase activity of the mutant reporter was unaffected by the simultaneous infection with miR-23a (Figure 4C). The results indicated that miR-23a may suppress gene expression through the miR-23a binding sequence in the 3′-UTR of PAK6.

The effects of miR-23a on the endogenous expression of PAK6 were further examined by Western blotting (Figure 4D). The exogenous overexpression of miR-23a in the PC-3 cells resulted in a marked decrease in PAK6 expression (79%), whereas miR-23a inhibitor oligonucleotides induced a pronounced increase in PAK6 expression (40%). These data suggested that miR-23a inhibited PAK6 expression at the post-transcriptional level by directly targeting the 3′-UTR of PAK6 mRNA.

### MiR-23a levels were inversely correlated with PAK6 protein levels in prostate cancer tissues

To examine whether the biological effects of down-regulating miR-23a correlated with PAK6 protein levels in clinical prostate cancer tissues, PAK6 protein levels in 20 paired prostate cancer and adjacent non-tumor tissues were examined by Western blotting, and miR-23a expression was determined by real-time PCR (Supplementary Figure 1A, 1B; the same samples as in Figure 1C). The extent of PAK6 up-regulation inversely correlated with degree of miR-23a down-regulation (R^2^ = 0.69, *P* < 0.05) (Figure 4E), suggesting that the inhibitory effects of miR-23a on PAK6 were clinically relevant in prostate cancer.

### PAK6 rescued effects of miR-23a on migration and invasion in prostate cancer cells

To determine whether PAK6 was involved in the miR-23a-mediated inhibition of migration and invasion in prostate cancer cells, we transfected PC-3 cells with siRNA-PAK6 (siPAK6). PAK6 knockdown by siRNA suppressed the migration and invasion of the PC-3 and DU145 cells (Figure 4F). We also performed rescue experiments by introducing a constitutively active form of PAK6 into PC-3, DU145, C2-4 and C4-2B cells. The transwell migration assays and matrigel invasion assays demonstrated that PAK6 overexpression reversed the miR-23a-mediated inhibition of migration and invasion in PC-3, DU145, C2-4 and C4-2B cells (Figure 4F). These data indicated that PAK6 was involved in the miR-23a-mediated inhibition of migration and invasion in prostate cancer cells.

### Ectopic expression of miR-23a impaired cytoskeletal events

**Figure 5:**
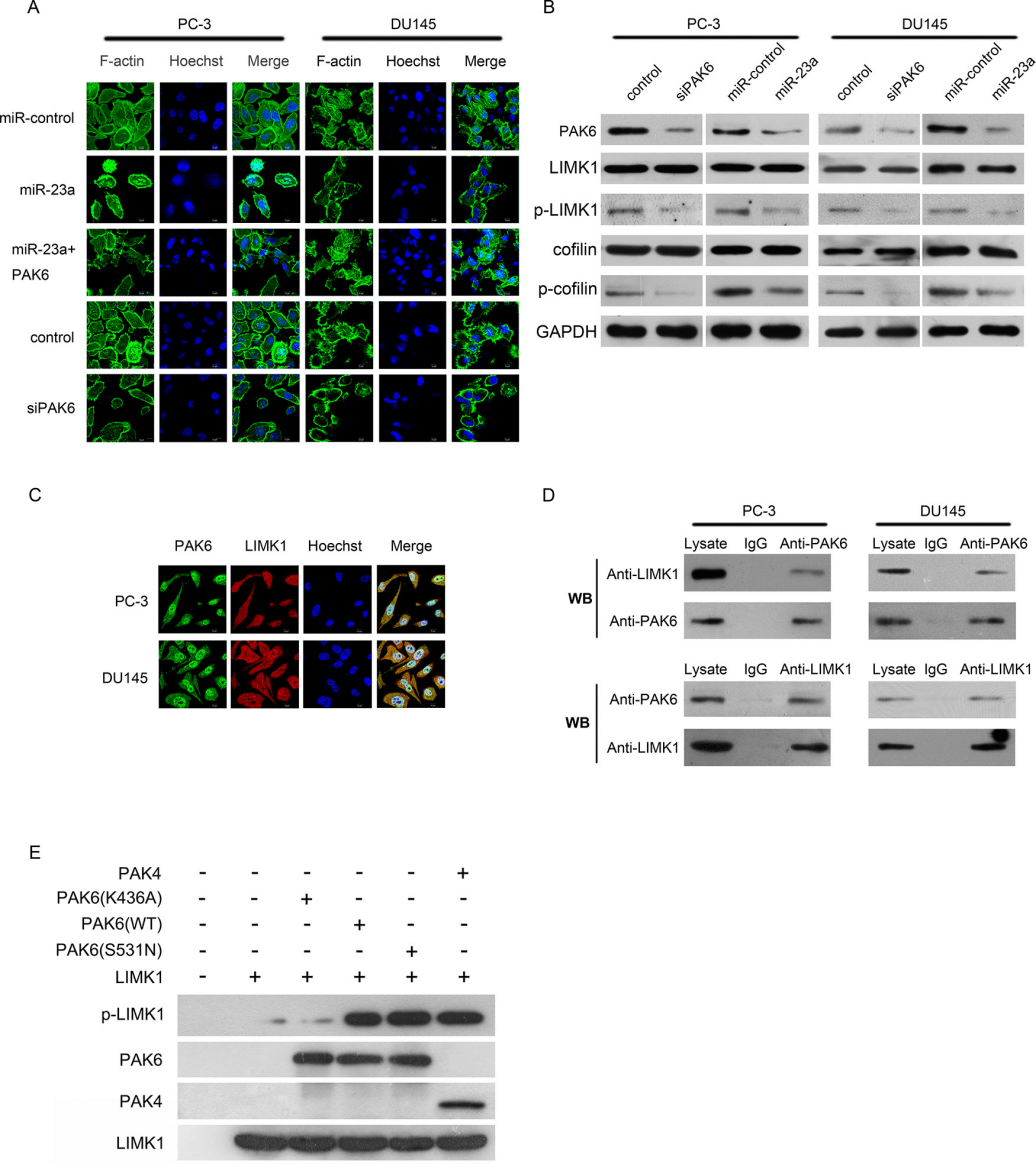
Mechanism by which miR-23a suppresses migration and invasion of prostate cancer cells **(A)** Ectopic expression of miR-23a or of si-PAK6 disrupted stress fiber network. Stress fibers and actin filaments were visualized by phalloidin staining (green). Scale bar: 10 μm. **(B)** Immunoblotting of PAK6, LIMK1, phosphorylated LIMK1 (p-LIMK1), cofilin and phosphorylated cofilin (p-cofilin) in PC-3 and DU145 cells infected respectively with si-PAK6, si-control, miR-control-lentivirus or miR-23a-lentivirus. **(C)** Immunofluorescent staining for PAK6 (green) and p-LIMK1 (red) in PC-3 cells (upper panels) or in DU145 cells (lower panels). Nuclei were counterstained with Hoechst33258 (blue). The right side illustrates merged images of PAK6, phosphorylated LIMK1 (p-LIMK1), and Hoechst staining. *Scale bar*: 10 μm. **(D)** PC-3 cell lysates were immunoprecipitated with PAK6 antibody and subjected to Western blotting to ascertain LIMK1 and PAK6 expression using indicated antibodies (upper panels). PC-3 cell lysates were immunoprecipitated with LIMK1 antibody and subjected to Western blotting using indicated antibodies (lower panels). **(E)** *In vitro* kinase assay was performed using purified PAK6 (K436A), PAK6 (S531N), PAK6 (WT), PAK4 and LIMK1 protein. Phosphorylated LIMK1 (p-LIMK1), PAK6, PAK4 and LIMK1 were measured by Western blotting.

The actin cytoskeleton is a dynamic structure, in which actin polymerization and depolymerization rates control cell motility, cell division, and the formation of specialized structures [23, 24]. To investigate the changes occurring in the actin cytoskeleton, we labeled the PC-3 and DU145 cells with phalloidin. Upon examination by laser confocal microscopy, we found that the dissolution of actin stress fibers and the formation of actin fibers at the cell peripheries were suppressed in the miR-23a-PC-3 cells and miR-23a-DU145 cells; similar results were observed in the PC-3 and DU145 cells transfected with siPAK6 (Figure 5A).

Cofilin, which is a substrate of LIMK1, plays an important role in promoting actin polymerization and defining the direction of cell motility [25, 26], we investigated whether LIMK1 and cofilin were involved in the inhibition of migration by miR-23a-PAK6. Firstly, PC-3 and DU145 cells were infected with siPAK6. Western blotting indicated that PAK6 siRNA knockdown in the PC-3 and DU145 cells markedly reduced the phosphorylation of LIMK1 and cofilin, whereas the total expression levels of LIMK1 and cofilin did not change (Figure 4B). Then, PC-3 and DU145 cells were infected with a miR-control-lentivirus or a miR-23a-lentivirus. Western blotting revealed that the phosphorylation of LIMK1 and cofilin was markedly reduced in the miR-23a-PC-3 and miR-23a-DU145 cells, whereas the total expression of LIMK1 and cofilin did not significantly change (Figure 5B). Together, these data suggested that LIMK1-cofilin signaling played an important role in the regulation of prostate cancer cell migration by miR-23a.

### PAK6 interacted with LIMK1 in prostate cancer cells

To investigate the possibility that PAK6 phosphorylated LIMK1 *in vivo*, we examined whether PAK6 physically interacted with LIMK1 in the PC-3 and DU145 cells. As shown by immunofluorescent staining, PAK6 (green) and LIMK1 (red) were predominantly localized within the cytoplasm (Figure 5C). The degree of co-localization (represented by yellow staining) was quantified using Pearson's correlation coefficient; the mean ± SE of PAK6 and LIMK1 colocalization was 0.79 ± 0.02 (*n* = 30) in the PC-3 cells and 0.76 ± 0.02 (*n* = 30) in the DU145 cells (complete co-localization was indicated by *a* value of 1.0). These data suggested that PAK6 and LIMK1 generally co-localized in the PC-3 and DU145 cells

To obtain biochemical evidence for the interaction between PAK6 and LIMK1, co-IP experiments were performed in the PC-3 and DU145 cells. The results showed that PAK6 interacted specifically with LIMK1 in the PC-3 and DU145 cells (Figure 5D). PC-3 cell lysates were incubated with a PAK6 antibody, and the immunocomplex was purified, separated by SDS-PAGE, and subjected to immunoblotting with an LIMK1 antibody. LIMK1 was present in the complex immunoprecipitated with the LIMK1 antibody (Figure 5D, upper panel). In addition, PAK6 was also present in the complex in a reciprocal immunoprecipitate formed using the antibody against LIMK1 (Figure 5D, lower panel). Neither PAK6 nor LIMK1 was detected in the immunocomplex in association with control IgG, validating the specificity of the observed co-association. These data indicated that PAK6 interacted with LIMK1 in PC-3 cells.

### PAK6 phosphorylated LIMK1 *in vitro*

To determine whether PAK6 directly phosphorylated LIMK1, an *in vitro* kinase assay was performed using purified kinase-defective PAK6 (K436A), activated PAK6 (S531N), wide type PAK6 (WT), wide type PAK4 (WT), and LIMK1 protein. The results showed that LIMK1 phosphorylation in the presence of PAK6 (K436A) was lower than that in the presence of PAK6 (WT) or PAK6 (S531N) (Figure 5E). These data indicated that PAK6 directly phosphorylated LIMK1 at Thr-508.

## DISCUSSION

The molecular mechanism by which miRNAs modulates cytoskeleton changes and metastasis has not been fully elucidated [27, 28]. Here, we demonstrated that miR-23a expression was specifically diminished in prostate cancer cell lines and human prostate cancer tissues. Low miR-23a expression was associated with a more aggressive tumor phenotype and was an independent predictor of reduced survival time in patients with prostate cancer. We also showed that miR-23a suppressed the migration and invasion of prostate cancer *in vitro* and tumor metastasis *in vivo*.

Previous studies showed that miR-23a is up-regulated in many types of cancer and is an important oncogene that promotes proliferation, migration and invasion and suppresses apoptosis. There was also one study from our research group reported that miR-23a is down-regulated in non-small cell lung cancer and suppresses the migration [29]. MiR-23a was also reported to be able to regulate the metabolism of tumor cells [30]. The c-Myc-mediated suppression of miR-23a/b enhances mitochondrial glutaminase expression and glutamine metabolism in prostate cancer. In the present study, we also found the function of miR-23a as a tumor suppressor in prostate cancer. MiRNA is able to exhibit diverse functionalities in different types of cancer cells. For example, miR-125b is multi-faceted, exhibiting tumor suppressor or oncogene activity depending on the cellular context [31]. Our findings suggested that miR-23a may be cancer type-specific, and play a role in the regulation of prostate cancer metastasis and invasion.

The invasion-metastasis cascade involves the dissemination of cancer cells to anatomically distant organ sites and their subsequent adaptations to foreign tissue microenvironments [32]. Actin polymerization generates protrusive activity at the front (anterior) of the cancer cell and, coupled with actin: myosin filaments generating contraction at the sides and the rear (posterior) of the cell, provides the major driving force for migration [33, 34]. In this study, the exogenous expression of miR-23a in the PC-3 and DU145 cells suppressed the dissolution of actin stress fibers and the formation of actin fibers at the cell peripheries; its target gene PAK6 counteracted the cytoskeletal changes.

PAK6 is a member of the PAK family, which is a growing class of Rac/Cdc42-associated Ste20-like Ser/Thr protein kinases that is characterized by a highly conserved amino-terminal Rac/Cdc42 interactive binding (CRIB) domain and a carboxy-terminal kinase domain [35]. PAKs are either up-regulated or hyper-activated in various human cancers, such as breast, ovarian, colorectal, thyroid, pancreatic and prostate cancer [36–38]. PAK6 is highly expressed in testis and prostate tissues. Our previous study showed that PAK6 is up-regulated in prostate cancer and that the knockdown of p21-activated kinase 6 inhibits prostate cancer cell migration [39]. To our knowledge, there have been no studies on the negative regulation of PAK6 by miRNA. In this study, we found that the relationship between PAK6 overexpression and miR-23a levels in prostate cancer tissues was negatively correlated. Subsequently, our results showed that miR-23a bound the complementary sites in the 3′-UTR of PAK6 and markedly decreased PAK6 protein expression. MiR-23a suppressed prostate cancer migration and invasion by directly targeting PAK6.

Cofilin is a small protein that freely diffuses within cells and can be found in multiple cellular compartments. Recent studies have confirmed that its activity is required for tumor cell motility and invasion [25, 40]. The local activation of cofilin by uncaging induces lamellipodia formation and dictates the direction of cell motility [37]. Cofilin is the only known physiological substrate of LIMK1 [41].

A potentially important observation in this study was that PAK6 activated LIMK1 activity by directly binding to LIMK1 and phosphorylating it at Thr-508. Furthermore, we discovered that PAK6 and LIMK1 co-localized in the PC-3/DU145 cells. These data suggested that the cytoskeletal changes regulated by PAK6 were mediated by LIMK1 and cofilin. PAK6 has been reported to be an important tumor suppressor in prostate cancer cells based on its ability to phosphorylate the androgen receptor [42, 43], tumorigenic E3 ligase murine double minute-2 (Mdm2) [43], and β-catenin [44]. In the present study, we showed that LIMK1 was a novel substrate of PAK6.

It was reported that PAK6/IQGAP1/E-cadherin/beta-catenin is a signal pathway affecting adherens [45]. Our experiments showed that in prostate cancer cells, the expression levels of E-cadherin and beta-catenin didn't change no matter LIMK1 was knockdown or over-expressed (data not shown). Furthermore, co-IP experiment also showed that LIMK1 did not interacted with IQGAP1, E-Cadherin or beta-catenin. These results suggested that PAK6-LIMK1 signaling might not cross act with PAK6/IQGAP1/E-cadherin axis.

In conclusion, we found that miR-23a-PAK6-LIMK1 regulatory pathway may contribute to prostate cancer metastasis. Potential therapeutic approach by targeting miR-23 is suggested.

## MATERIALS AND METHODS

### Tissue specimens

Prostate cancer and adjacent normal tissue samples were obtained with informed consent in accordance with institutional review board-approved protocols. Twenty paired prostate cancer and adjacent matched non-tumor tissues and 10 metastatic prostate cancer samples were collected between August 2009 and June 2010 at the Third Affiliated Hospital, Sun Yat-sen University (Guangzhou, China). The fresh tissue samples were immediately snap-frozen in liquid nitrogen. In total, 123 prostate cancer tissues were collected between August 2003 and August 2008. All the diagnoses were histopathologically confirmed. The median follow-up time of the 123 patients with prostate cancer was 72 months (range, 60–120 months). The tumor and noncancerous samples were both histologically confirmed. This study was approved by the research ethics committee at the institute.

### Cell lines and cell culture

The following cell lines were used in this study: PC-3, DU145, LNCaP, C4-2, C4-2B, RWPE-1 and 293T. The RWPE-1 cell line was cultured in keratinocyte growth medium supplemented with 5 ng/ml human recombinant epidermal growth factor and 0.05 mg/ml bovine pituitary extract (Invitrogen Life Technologies, CA, USA). The prostate cancer cell lines (LNCaP, C4-2, C4-2B, DU145 and PC-3) and 293T cells were maintained in RPMI 1640 media (Gibco, Invitrogen Life Technologies, CA, USA) supplemented with 10% newborn calf serum (Gibco, Invitrogen Life Technologies, CA, USA).

### RNA isolation and quantitative real-time PCR

Total RNA was extracted using TRIzol reagent (Invitrogen Life Technologies, CA, USA). cDNA was synthesized with the PrimeScript RT Reagent Kit (Promega, Madison, WI). Real-time PCR was performed using the ABI 7900HT Fast Real-Time PCR system (Applied Biosystems, CA, USA). The following primers were used: PAK6 forward 5′-GACTCCATCCTGCTGACCCTC-3′ and reverse 5′-CACCTCAGTGGCATACAAAGACC-3′; miR23a forward 5′-ATCACATTGCCAGGGATTTCC-3′ and reverse 5′-CCAGTGCAGGGTCCGAGGT-3′; beta-actin forward 5′-TGACGTGGACATCCGCAAAG-3′ and reverse 5′-CTGGAAGGTGGACAGCGAGG-3′; and U6 forward 5′-TGCGGGTGCTCGCTTCGGCAGC-3′ and reverse 5′-CCAGTGCAGGGTCCGAGGT-3′.

### MiRNA profiling by miRCURY LNA universal RT miRNA PCR

Isolation of RNA and all real-time quantitative PCR (Q-PCR) experiments were performed according to the protocol of MiRNA profiling by miRCURY LNA Universal RT miRNA PCR (Exiqon). MiRNA microarray analysis performed by KangChen Corporation (Shanghai, China) was used to compare the miRNA expression profiles in three pairs of prostate cancer and matched adjacent normal tissues.

### Vector construction

The pre-miR-23a and pre-miR-23a-sponge-inhibitor sequences were synthesized and cloned into pGLV-H1-GFP-Puro (GenePharma, Shanghai, China) to generate the pGLV-H1-GFP-Puro-miR-23a and pGLV-H1-GFP-Puro-miR-23a-inhibit expression vectors, respectively. The PAK6 and pGLV4-EF1α-EGFP-luciferase plasmids were purchased from GenePharma (Shanghai, China).

### Lentivirus production and transduction

Virus particles were harvested 48 h after the pGLV-H1-GFP-Puro-miR-23a, pGLV-H1-GFP-Puro-inhibitor and pGLV4-EF1a-EGFP-luciferase were transfected along with the packaging plasmids, PG-P1-VSVG, PG-P2-REV and PG-P3-RRE, into 293T cells using Lipofectamine 2000 (Invitrogen Life Technologies, Carlsbad, CA). The PC-3 and DU145 cells were infected with recombinant lentivirus-transducing units and 5 mg/ml Polybrene (Sigma-Aldrich, St Louis, Missouri).

### Oligonucleotide transfection

A miR-23a mimic and miR-23a inhibitor (anti-miR-23a, chemically modified antisense oligonucleotides designed to specifically target mature miR-23a) were synthesized by GenePharma (Shanghai, China). PAK6 siRNA was designed and synthesized by Santa Cruz Biotechnology (California, USA). The oligonucleotides were transfected using the siPOR™ NeoFX™ Transfection Agent (10 μl in 200 μl of OPTI-MEM I medium without serum) for 5 min. The final concentration of miR-23a mimic in the transfection system was 50 nM, and the final concentration of anti-miR-23a in the transfection system was 200 nM.

### Luciferase reporter assay

The expression vector for miR-23a (pc3-miR-23a) was generated by cloning genomic fragments encompassing the miR-23a precursor and its 5′- and 3′-flanking sequences into pcDNA3.0 (Invitrogen Life Technologies, Carlsbad, CA). The plasmid pc3-gab was produced based on pcDNA3.0 by replacing the neomycin open reading frame with an enhanced green fluorescent protein (EGFP) expression cassette. The PAK6 coding sequence was cloned into pc3-gab to generate an expression vector (pc3-gab-pak6).

To create a luciferase reporter construct, a 3cDNA3.0 by replacing the neomycin open reading frame with an enhanced green fluorescent protein (EGFP) expression cassette. The PAK6 coding sequence was cloned into pc3-gab to generate ahe complementary site for the seed region of miR-23a was generated using the fusion PCR method. 293T cells grown in a 48-well plate were cotransfected with 200 ng of pcDNA3.0 or 200 ng of pc3-miR-23a, 10 ng of firefly luciferase reporter containing the wild-type or mutant 3′-UTR of the target gene, and 2 ng of pRL-TK (Promega, Madison, WI).

### Matrigel invasion assays and transwell migration assays

For the Matrigel invasion assays, 5 × 10^4^ cells were added to a matrigel invasion chamber (BD Biosciences, CA, USA) in an insert of a 24-well culture plate. FBS was added to the lower chamber as a chemoattractant. After 24 h, the non-invading cells were gently removed with a cotton swab. Invasive cells located on the lower side of the chamber were stained with crystal violet, air-dried and photographed. The Transwell migration assays were performed in a similar manner as the Matrigel invasion assays but without Matrigel on the filter. All experiments were performed in triplicate and were repeated once.

### Western blotting

Western blotting was performed as described previously [46].

### Actin polymerization assays

Control and pSuper-miR-23a-transfected PC-3 cells and DU145 cells were seeded on fibronectin-pretreated chamber slides. The cells were fixed with glutaraldehyde for 10 min at room temperature and then washed three times with 1% BSA for 5 min. The cells were then treated with 0.25% Triton X-100, 1% BSA, and 10% normal donkey serum in PBS at room temperature for 45 min. Phalloidin was purchased from Sigma-Aldrich. Tumor cells were incubated with rhodamine phalloidin for 1 h at 37°C. The cells were then washed three times with PBS. Images of the cells were captured using confocal laser scanning microscopy.

### Immunoprecipitation (IP)

IP assays were performed as described previously [46]. PC-3 cells (6 × 10^6^) were solubilized in 400 μl of cell lysis buffer (1% Triton X-100, 150 mm NaCl, 20 mm Tris-Cl (pH 7.4), 1 mm EDTA, 1 mm EGTA, 1 mm Na_3_VO_4_, 2.5 mm pyrophosphate, 1 mm glycerol phosphate, and a protease inhibitor mixture) for 10 min at 4°C. After brief sonication, the lysates were cleared by centrifugation at 15,000 × *g* for 10 min at 4°C, and the cell extract was immunoprecipitated with 4 μg of PAK6 (Merck) or LIMK1 (Cell Signaling) antibody and then incubated with 60 μl of protein G plus/protein A-agarose for 16 h at 4°C by continuous inversion. The immunocomplexes were pelleted and washed three times. The precipitated immunocomplexes were then boiled in Laemmli buffer and subjected to Western blotting with an anti-LIMK1 or an anti-PAK6 antibody.

### Immunofluorescence

PC-3 and DU145 cells were grown on cover glasses, fixed using freshly prepared 4% paraformaldehyde, and permeabilized with 0.1% Triton X-100 in TBS. The cover glasses were incubated with the primary antibodies (anti-PAK6, Santa Cruz Biotechnology; anti-LIMK1, Cell Signaling Technology) at 1:50 dilutions. PAK6 was detected with an anti-goat secondary antibody conjugated to Alexa Fluor 488 (Invitrogen Life Technologies). LIMK1 was detected with an anti-rabbit secondary antibody conjugated to Alexa Fluor 555 (Invitrogen Life Technologies). The fluorescent staining was visualized using a 63 × NA 1.3 oil objective on a confocal microscope (LSM 510 Meta; Carl Zeiss, Inc.). The images were recorded with sequential acquisition settings at a resolution of 512 × 512 pixels with a 12-bit depth. In addition, a 420-480 nm bandpass emission filter was used for the blue channel, a 560 nm long-pass filter was used for the red channel, and a 505-530 nm bandpass emission filter was used for the green channel. The confocal settings were fixed for the duration of the experiments to enable the comparisons of the fluorescence intensities. The images were processed using LSM 510 software (Carl Zeiss, Inc.) before being imported into Photoshop CS2 (Adobe) for orientation and cropping. The co-localization of PAK6 with LIMK1 was estimated using Pearson's correlation coefficient (full co-localization = 1.0) using the Image Pro Plus software.

### Protein kinase assay

PAK6 (WT) cDNA was cloned into the pET30a *E. coli* expression vector, and the mutant vectors PAK6 (K436A) and PAK6 (S531N) were created by site-directed mutagenesis. Recombinant PAK6 (K436A), PAK6 (S531N), PAK6 (WT) and, PAK4 proteins were prepared and purified from *E. coli* expression systems. LIMK1 protein was obtained from Life Technologies (Carlsbad, CA, USA). Equal amounts of the fusion proteins were incubated in buffer containing 50 mM HEPES (pH 7.5), 10 mM MgCl_2_, 100 mM NaCl, 1 mM DTT, and 50 μM ATP for 30 min at 30°C. The reaction was terminated by the addition of 3 × SDS sample buffer. LIMK1 phosphorylation at Thr-508 was examined by Western blotting.

### Orthotopic prostate cancer model by PC-3 cells

PC-3 cells stably expressing either empty vector or miR-23a and a luciferase reporter were generated by retroviral transduction. Under anesthesia, 1 × 10^6^ PC-3-Luc cells were injected into the prostates of 5-week-old male athymic nude mice. Bioluminescence imaging was performed on the mice at 7-d intervals using a charge-coupled device camera (IVIS; Xenogen Corp). The data were analyzed using the IVIS Living Image software (Xenogen Corp). The mice were killed by cervical dislocation 56 d after implantation. An autopsy was performed on the orthotopic animal models to assess the distribution of metastases. The prostate tumors and metastases were harvested and fixed in 10% formalin for histological analyses. The animal handling and experimental procedures were approved by the Medicine Institutional Animal Care and Use Committee of the Third Affiliated Hospital, Sun Yat-sen University.

### Statistical analysis

The data are presented as the mean ± SE from at least three independent experiments. All statistical analyses were performed using the SPSS software (version 17.0). The differences between variables were assessed using the χ^2^ test. For the survival analysis, we analyzed all patients with prostate cancer by Kaplan-Meier analysis. Survival differences were analyzed with the log-rank test. Multivariate survival analysis was performed on all significant parameters from the univariate analysis using the Cox regression model. *P* values < 0.05 were considered to be significant.

## SUPPLEMENTARY TABLE AND FIGURE


