# Supplementary material for: Downregulation of microRNA-23a suppresses prostate cancer metastasis by targeting the PAK6-LIMK1 signaling pathway
Source: Oncotarget. 2015 Jan 16;6(6):3904–17. doi: 10.18632/oncotarget.2880 (PMC4414162; doi:10.18632/oncotarget.2880)
Supplement: Supplementary file 1 [file oncotarget-06-3904-s001.pdf]

## SUPPLEMENTARY TABLE AND FIGURE

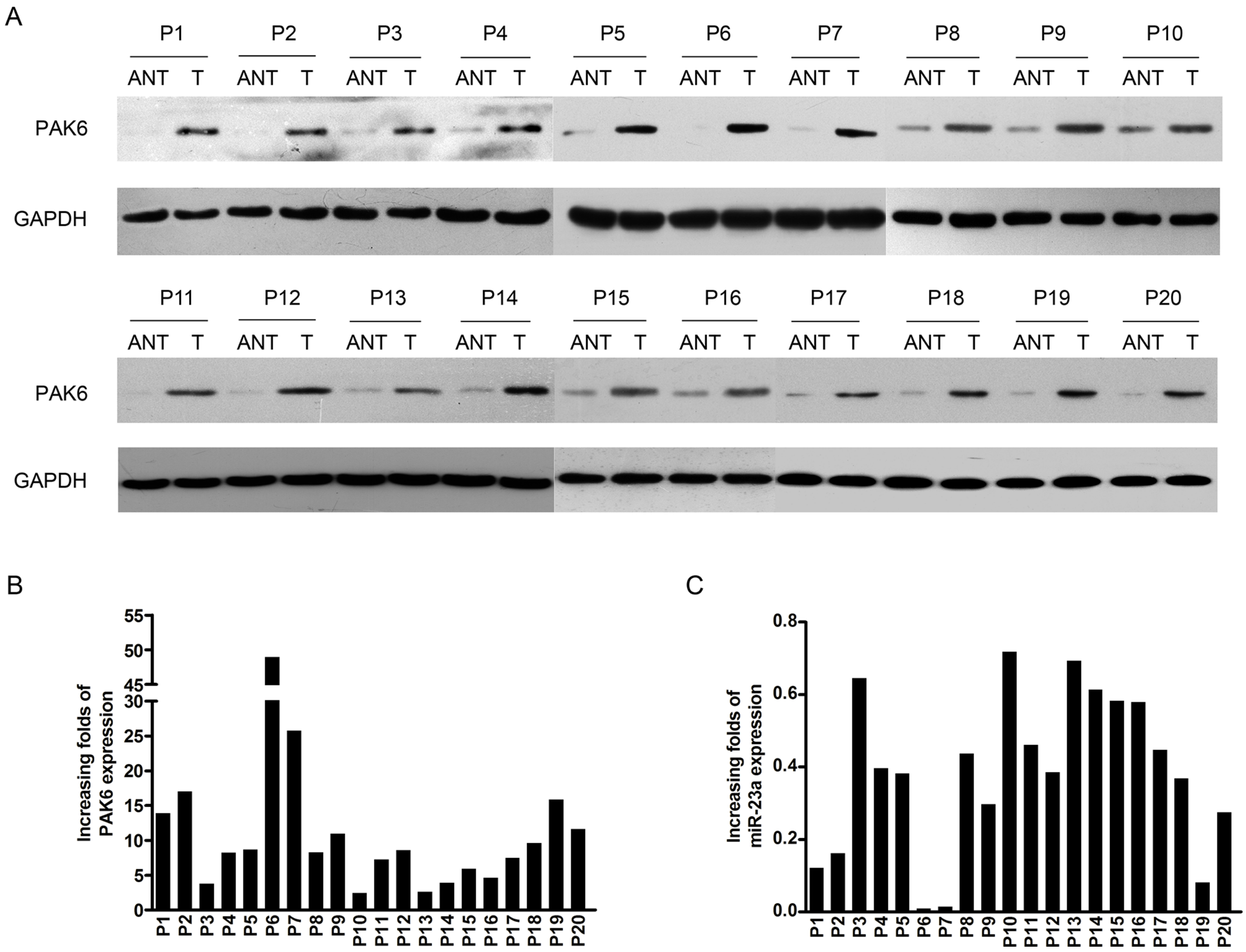


**Supplementary Figure 1: Clinical relationship between miR-23a and PAK6 expression in human prostate cancer tissues. (A)** Western blotting for PAK6 protein expression in aforementioned 20 pairs of surgically resected fresh prostate cancer tissues. GAPDH was used as loading control. **(B)** PAK6 protein expression was quantified by scanning densitometry and scored. Fold increase in PAK6 expression in prostate cancer tissues compared with matched adjacent non-cancerous tissues was calculated for each patient. **(C)** Fold increase in miR-23a expression in prostate cancer tissue compared with matched adjacent non-cancerous tissues was calculated for each patient.

**Supplementary Table 1: 51 microRNAs were identified as altered markedly in three pairs of primary prostate cancer and matched adjacent non-tumor tissues by examining the expression profiling of human miRNAs**

| miRNA ID | *P* Value | Fold Up- or Down-Regulation | miRNA ID | *P* Value | Fold Up- or Down-Regulation |
|---|---|---|---|---|---|
| miR-328 | 0.0312 | −7.21 | miR-432 | 0.0029 | −4.58 |
| miR-143 | 0.0433 | −27.52 | miR-337-3p | 0.0353 | −7.24 |
| miR-218 | 0.0490 | −9.97 | miR-144 | 0.0398 | 5.99 |
| miR-140-5p | 0.0394 | −3.65 | miR-184 | 0.0148 | −27.42 |
| miR-30c | 0.0419 | −5.46 | let-7c | 0.0084 | −7.22 |
| miR-487b | 0.0402 | −8.26 | miR-488 | 0.0032 | −200.36 |
| miR-130a | 0.0269 | −9.04 | miR-455-5p | 0.0199 | −5.91 |
| miR-26a-2* | 0.0200 | −8.27 | miR-30e* | 0.0166 | −5.83 |
| miR-221 | 0.0156 | −17.65 | miR-181c | 0.0111 | −7.58 |
| miR-424 | 0.0031 | −4.81 | miR-499-5p | 0.0378 | −17.95 |
| miR-532-5p | 0.0218 | −3.11 | miR-152 | 0.0144 | −12.05 |
| miR-99a | 0.0121 | −5.55 | miR-29c | 0.0155 | −9.90 |
| miR-125b | 0.0215 | −7.77 | miR-133a | 0.0275 | −41.76 |
| miR-574-3p | 0.0446 | −4.90 | miR-299-5p | 0.0260 | −6.43 |
| let-7e | 0.0297 | −5.75 | miR-145 | 0.0373 | −26.33 |
| miR-133b | 0.0494 | −32.50 | miR-29a | 0.0270 | −9.00 |
| miR-204 | 0.0406 | −69.06 | miR-222 | 0.0174 | −15.01 |
| miR-374b | 0.0299 | −4.12 | miR-181b | 0.0290 | −4.55 |
| miR-26a | 0.0225 | −12.07 | miR-1 | 0.0271 | −36.74 |
| miR-99b | 0.0237 | −5.52 | miR-338-3p | 0.0459 | −5.26 |
| miR-23a | 0.0428 | −13.81 | miR-216a | 0.0461 | 92.97 |
| miR-505 | 0.0321 | −3.79 | miR-450a | 0.0188 | −5.49 |
| miR-887 | 0.0494 | −4.87 | miR-365 | 0.0426 | −5.70 |
| miR-873 | 0.0040 | −125.68 | miR-100 | 0.0252 | −11.85 |
| miR-181d | 0.0402 | −7.61 | miR-27b | 0.0118 | −9.32 |
| miR-342-3p | 0.0257 | −4.03 | | | |
